# Emerging copper-based hollow nanoplatforms for cancer treatment

**DOI:** 10.1016/j.ijpx.2026.100608

**Published:** 2026-07-11

**Authors:** Shuang Liang, Xingran Li, Li Zhang, Wenzhen Zhu, Shaoyan Gan

**Affiliations:** a Department of Radiology, Tongji Hospital, Tongji Medical College, Huazhong University of Science and Technology, Wuhan 430030, People's Republic of China; b State Key Laboratory of New Textile Materials and Advanced Processing, Wuhan Textile University, Wuhan 430200, People's Republic of China; c Lucky Huaguang Graphics Co., Ltd, Nanyang 473000, People's Republic of China; d School of Food Science and Engineering, Wuhan Polytechnic University, Wuhan 430023, People's Republic of China

**Keywords:** Copper, Hollow nanoplatforms, Chemodynamic therapy, Cuproptosis, Synergistic therapeutic outcomes

## Abstract

Nanomedicines have significantly advanced tumor treatment, with hollow copper (Cu)-based nanostructures emerging as a uniquely promising nanoplatform. Their degradable frameworks enable controlled release of various therapeutic agents, facilitating chemotherapy, photothermal therapy (PTT), photodynamic therapy (PDT), immunotherapy, and related modalities. Simultaneously, liberated Cu ions drive chemodynamic therapy (CDT) via Fenton-like reactions and trigger cuproptosis. A critical effect of this combined action is the induction of immunogenic cell death (ICD), thereby amplifying antitumor immunity. Moreover, the inherent physicochemical properties of these nanostructures support multimodal diagnostic functions, which enable accurate imaging-guided interventions. Herein, we present a systematic overview of the latest developments in hollow Cu-based nanoplatforms, covering inorganic Cu nanostructures, Cu-doped composites, matrix-supported Cu systems, and Cu-based complexes, for diverse cancer therapies. We also discuss rational design strategies to achieve synergistic therapeutic outcomes and conclude with an analysis of translational challenges and future directions toward clinical adoption.

## Highlights

• First systematic review of hollow Cu-based nanoplatforms for cancer therapy.
• Cutting-edge strategies for cuproptosis/CDT-based multimodal treatment.
• Critical insights into challenges and prospects of hollow Cu-based nanomedicines.

## Graphical abstract

**Figure:**
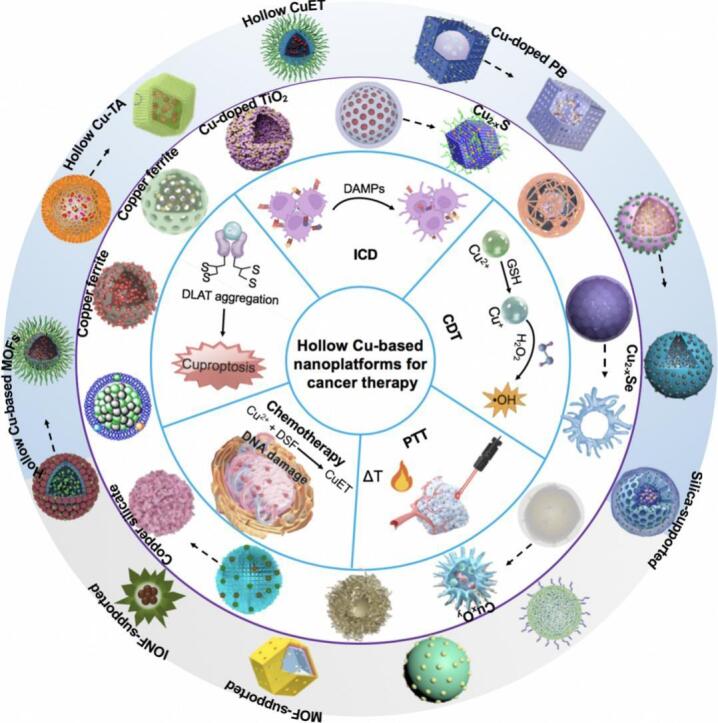

## 1 Introduction

Cancer remains a primary contributor to mortality worldwide. The efficacy of conventional therapies is often limited by challenges including a hypoxic tumor microenvironment (TME), multidrug resistance (MDR), and severe off-target effects (Liao et al., 2022; Man et al., 2025; Zhang et al., 2022a). These shortcomings underscore the urgent need for innovative and precise treatment modalities. In recent years, stimuli-responsive nanomedicines have emerged as a promising frontier in oncology. Designed to be activated by external triggers (e.g., near-infrared (NIR) light, ultrasound (US)) or intrinsic TME cues (e.g., acidic pH, glutathione (GSH), hydrogen peroxide (H_2_O_2_)), these systems enable spatiotemporally controlled drug release and therapeutic action (Huang et al., 2025; Liang et al., 2024b; Zhang et al., 2025e).

Among diverse nanomaterials, copper (Cu)-based systems have attracted considerable interest due to their unique physicochemical and versatile biological effects, making them ideal candidates for integrated theranostics (Li et al., 2023b). Unlike single-function agents, Cu-based nanoplatforms serve dual roles as contrast agents for photoacoustic/magnetic resonance imaging (PAI/MRI) and as potent therapeutic agents (Li et al., 2022; Zhang et al., 2025c). Copper chalcogenides (e.g., copper sulphide (CuS) and copper selenide (CuSe)) possess intense NIR absorption and high photothermal conversion efficiency (PCE), positioning them as excellent photothermal agents (PTAs) for tumor ablation (Li et al., 2024b; Ma et al., 2023). Copper oxides (e.g., copper oxide (CuO), cuprous oxide (Cu_2_O)) and copper-based metal-organic frameworks (Cu-MOFs) catalyze Fenton-like reactions in the TME, converting endogenous H_2_O_2_ into cytotoxic hydroxyl radicals (•OH) for chemodynamic therapy (CDT), a modality that inherently bypasses MDR (Feng et al., 2024a; Soman et al., 2025; Wang et al., 2022; Yang et al., 2023; Zhong et al., 2022). Beyond the phenomenological advantages of copper-based nanomaterials, quantitative kinetic considerations provide a compelling physicochemical rationale for their selection over traditional iron-based Fenton agents. Classical iron-based Fenton chemistry has a second-order rate constant of 40–80 M^−1^ s^−1^ for Fe^2+^ oxidation, with the rate-determining reduction of Fe^3+^ to Fe^2+^ proceeding at only 2.7 × 10^−3^ s^−1^, severely limiting overall efficiency. In contrast, the Cu^+^-mediated Fenton-like reaction exhibits a rate constant of approximately 4.1 × 10^3^ M^−1^ s^−1^ for Cu^+^ oxidation by H_2_O_2_—roughly two orders of magnitude higher than the iron-based system. This kinetic advantage arises from the more favorable redox potential of the Cu^+^/Cu^2+^ couple. Furthermore, in bimetallic Cu—Fe systems, Cu^+^ reduces Fe^3+^ back to Fe^2+^, sustaining long-term •OH production. Critically, copper-based Fenton-like reactions maintain high activity under mildly acidic conditions (pH 5–6.5) that mimic the TME whereas iron-based reactions are severely limited at pH > 3 due to Fe^3+^ precipitation. Furthermore, Cu-MOFs act as sonosensitizers or photosensitizers under US or light irradiation, generating reactive oxygen species (ROS) for sonodynamic (SDT) or photodynamic therapy (PDT) (Han et al., 2024; Hao et al., 2023; Xu et al., 2023a). Recently identified as a unique form of regulated cell death, cuproptosis results from intracellular copper overload, instigating mitochondrial protein aggregation and depletion of iron‑sulfur clusters. Distinct from apoptosis, ferroptosis, or pyroptosis, cuproptosis offers a promising route to overcome apoptosis resistance (Mao et al., 2025; Zhang et al., 2025g).

To fully exploit this theranostic potential, rational nanostructural design is paramount. Hollow nanostructures, characterized by a cavity and thin shell, have emerged as superior carriers. Their advantages include high cargo-loading capacity for therapeutic agents (e.g., chemotherapeutics, photosensitizers, immunomodulators), extensive surface area for multifunctional modification, and tunable shell permeability for controlled release (Liang et al., 2022; Liu et al., 2025c). Synthesis strategies such as hard/soft-templating, self-templating, and template-free methods allow precise control over size, shape, and porosity (El-Toni et al., 2016; Li et al., 2023c; Zhang et al., 2022b). Systematic comparison with non-hollow counterparts further substantiates the architectural superiority of hollow Cu-based nanoplatforms. The large internal cavity and mesoporous shell provide substantially higher surface area and void volume, enabling markedly greater co-loading of therapeutic agents than the limited surface adsorption available to solid Cu nanoparticles. The thin, defect-rich shell exposes abundant accessible catalytic sites, yielding considerably higher Fenton-like activity and ROS output. Hollow shells also undergo rapid TME-responsive disintegration, whereas compact solid crystals resist degradation and tend to persist in organs. This tumor-selective degradation profile minimizes off-target metal ion leakage and associated systemic toxicity. Consequently, hollow Cu nanosystems achieve superior multimodal synergistic efficacy over their solid analogues.

**Fig. 1:**
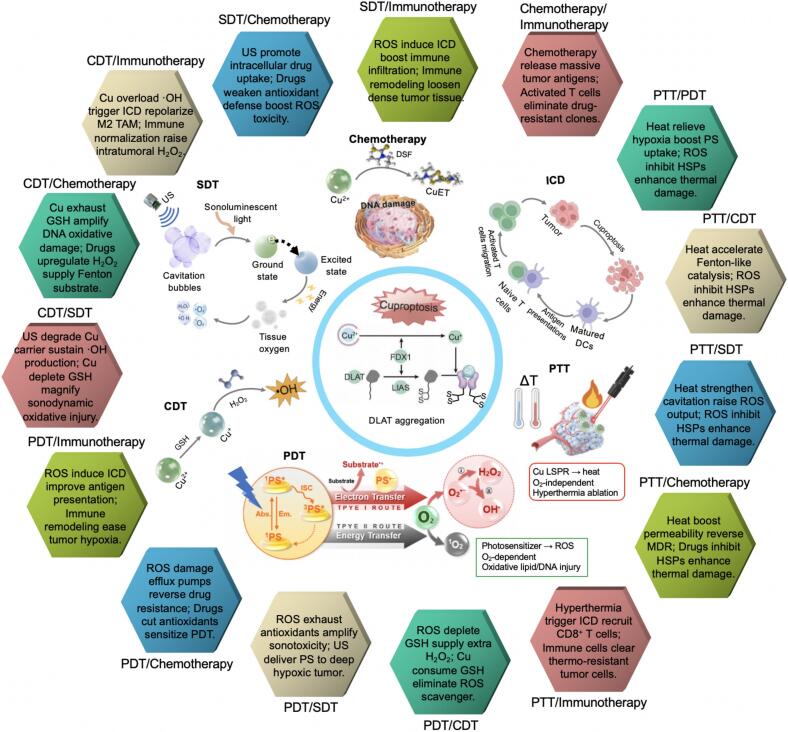
Comprehensive schematic depicting monotherapy pathways and mutual sensitization mechanisms of combinatorial multimodal tumor therapies constructed on hollow Cu-based nanoplatforms.

A critical advantage of hollow Cu-based nanosystems is their TME-responsive degradation. In the acidic, GSH-rich TME, the shell degrades controllably, releasing bioactive Cu^2+^/Cu^+^ and co-loaded therapeutics. This enables precise, localized treatment and initiates a self-amplifying cycle where the ions enhance CDT and induce cuproptosis (Deng et al., 2024; Mao et al., 2025). Importantly, this degradability promotes metabolic clearance, minimizing long-term toxicity. Beyond direct therapeutic effects, these hollow nanoplatforms can initiate an immunogenic cell death (ICD) cascade. This process converts dying malignant cells into endogenous vaccines through liberation of tumor-associated antigens and damage-associated molecular patterns (DAMPs). Such immunogenic signaling facilitates dendritic cell (DC) maturation and activation, which in turn prime tumor-specific cytotoxic T lymphocytes (CTLs), orchestrating a systemic antitumor immune response (Jing et al., 2025; Sensi et al., 2024). Modalities like PTT, CDT, and chemotherapy mediated by hollow Cu-based nanoplatforms have been shown to trigger ICD (Fan et al., 2025; Hu et al., 2024b; Li et al., 2025b). Their surfaces can also be functionalized with immunomodulators (e.g., checkpoint inhibitors, cytokines) to counteract TME immunosuppression, offering potential for durable tumor regression and metastasis prevention (Galluzzi et al., 2017; Hu et al., 2024c; Liang et al., 2024a; Zhang et al., 2025d). Collectively, hollow copper-based nanoplatforms integrate six mainstream tumor therapeutic modalities and build self-amplifying therapeutic feedback loops. The comprehensive pairwise bidirectional synergistic mechanisms of these combinatorial therapies are summarized in Fig. 1.

**Fig. 2:**
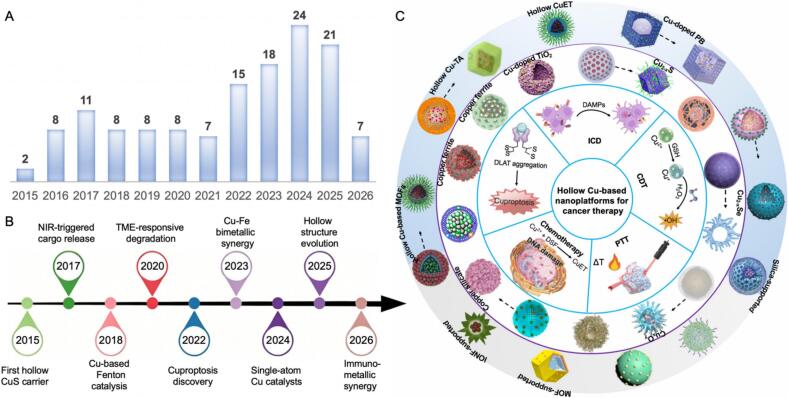
(A) Annual publication trend from 2015 to 2026 (Web of Science, keywords: “hollow copper” AND “cancer therapy”). (B) Chronological timeline of key milestones in the development of Cu-based nanoplatforms for cancer therapy from 2015 to 2026. (C) Schematic illustration of various hollow Cu-based nanoplatforms for cancer treatment.

This review comprehensively surveys the burgeoning landscape of hollow Cu-based nanoplatforms for cancer treatment. We systematically categorize them into inorganic, doped, matrix-supported, and complex-based hollow structures, detailing their synthesis, properties, and therapeutic applications. Particular focus is placed on the synergistic integration of CDT and cuproptosis with other treatment modalities (Fig. 2). Finally, we discuss translational challenges and future perspectives, aiming to inspire rational design and accelerate clinical application.

## 2 Hollow Cu-based inorganic nanoplatforms for cancer treatment

Hollow Cu-based inorganic nanoplatforms including copper chalcogenides, oxides, silicates, and bimetallic composites are the most extensively studied category in Cu-based theranostics. Their inherent properties, such as high PCE, excellent Fenton-like catalytic activity, and cuproptosis-inducing capability, render them suitable for multiple therapeutic modalities. Moreover, their hollow structure ensures high physiological stability while facilitating efficient cargo loading and TME-responsive degradation. This section summarizes recent advances in these nanoplatforms, focusing on structural design, therapeutic mechanisms, and synergistic effects.

### 2.1 Hollow Cu_2-x_S nanoplatforms

Hollow Cu_2-x_S nanoplatforms, typically synthesized from Cu_2_O via the Kirkendall effect, have attracted substantial interest due to their strong NIR absorption, high PCE, and good biocompatibility (Deng et al., 2017; Yang et al., 2024b). Recent efforts have focused on integrating CuS with other therapeutic units to overcome the limitations of single-modal therapy. Combining photothermal effects with cuproptosis induction has emerged as a promising strategy for enhancing anticancer immunity (Li et al., 2024a; Niu et al., 2025). However, translation remains challenging due to inefficient intracellular copper delivery, elevated intratumoral GSH levels, and potential off-target toxicity.

**Fig. 3:**
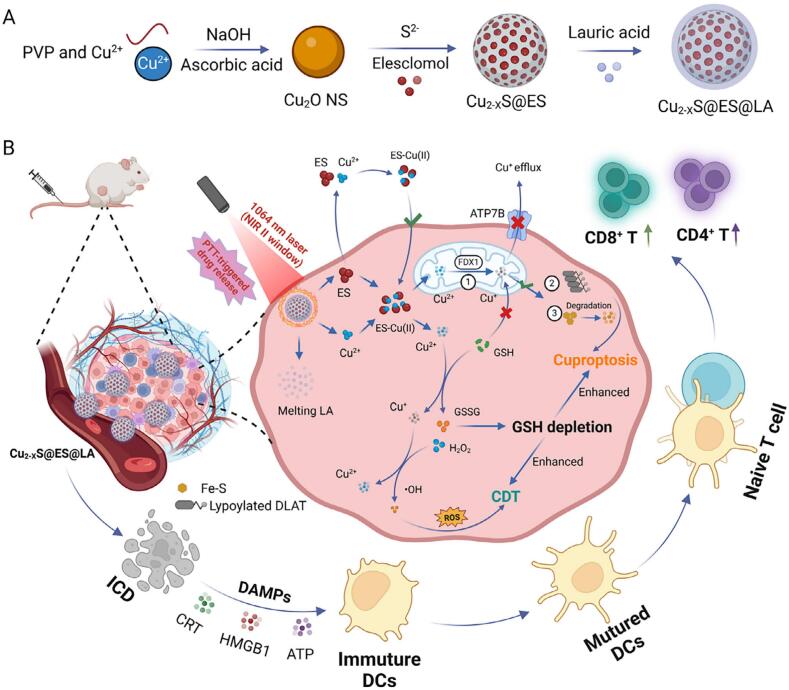
Schematic illustration of the NIR-II photothermal-triggered cuproptosis effect of CEL. (A) Synthesis process of CEL. (B) Mechanism of the therapeutic effect of amplified cuproptosis combined PTT/CDT and the activation of the immune response. Adapted with permission from ref. (Cheng et al., 2025), copyright 2025, Wiley-VCH GmbH.

To address these limitations, Cheng et al. (Cheng et al., 2025) designed a self-amplifying cuproptosis nanoplatform (Cu_2-x_S@ES@LA, CEL) composed of hollow Cu_2-x_S nanospheres, the copper ionophore elesclomol (ES), and a temperature-sensitive phase-change material, lauric acid (LA). Under NIR-II laser irradiation, Cu_2-x_S generates photothermal energy that melts LA, enabling controlled, tumor-localized release of ES and Cu^2+^. The released ES readily crosses cell membranes, forming ES–Cu^2+^ complexes that substantially enhance intracellular copper accumulation. Moreover, excess Cu^2+^ depletes GSH, alleviating its inhibitory effect on cuproptosis. The resulting amplified cuproptosis triggers ICD, stimulating a potent antitumor immune response and tumor regression. After CEL + NIR treatment, DC maturation (CD80^+^CD86^+^) increased to 28.2%, and substantial infiltration of CD3^+^CD8^+^ T cells (33.7%) was observed within tumors. Both values were significantly higher than those in other treatment groups. Conversely, the proportion of regulatory T cells (Tregs, CD4^+^FoxP3^+^) decreased to 9.85% after treatment, further alleviating immune suppression of CTLs. By enabling precise, on-demand delivery of both copper and its ionophore, the CEL nanoplatform offers a novel strategy for harnessing cuproptosis in cancer immunotherapy (Fig. 3).

Bimetallic sulfide nanoparticles are highly promising due to their enhanced catalytic activity for ROS generation, tunable electronic structures, and synergistic intermetallic effects for multimodal imaging (Guo et al., 2025; Xu et al., 2025). Jiang et al. (Jiang et al., 2022) reported a dually enhanced CDT strategy using polyethylene glycol (PEG)-functionalized bimetallic sulfide (Co_3-x_Cu_x_S_4_) NPs synthesized from zeolitic imidazolate framework-67 (ZIF-67). These NPs simultaneously inhibit •OH consumption (via GSH depletion) and promote •OH production (via hyperthermia acceleration). Similarly, Zhu et al. (Zhu et al., 2024b) constructed an intelligent nanoplatform (TPZ@Cu-SnS_2-x_/PLL) activated by both TME and NIR light. Cu doping and sulfur vacancies broaden the optical response, enhance charge carrier separation, and increase carrier density, endowing the system with optimal photothermal and photodynamic performance.

### 2.2 Hollow Cu_2-x_Se nanoplatforms

Similar to Cu_2-x_S, hollow Cu_2-x_Se nanoplatforms exhibit excellent NIR absorption and photothermal properties (Feng et al., 2024b; Wu et al., 2026). Yin et al. (Yin et al., 2025) synthesized sea urchin-like manganese-doped copper selenide nanoparticles (Mn-Cu_2-x_Se NPs) via an anion exchange method. These NPs were loaded with doxorubicin (DOX) and encapsulated within a protective bilayer of calcium alginate and chitosan (AC) to form DOX/Mn-Cu_2-x_Se@AC capsules. Their unique morphology enhances NIR-II absorption for effective PTT. Furthermore, Mn doping augments Fenton-like reactions for CDT, while NIR irradiation triggers DOX release, enabling a synergistic therapeutic regimen.

**Fig. 4:**
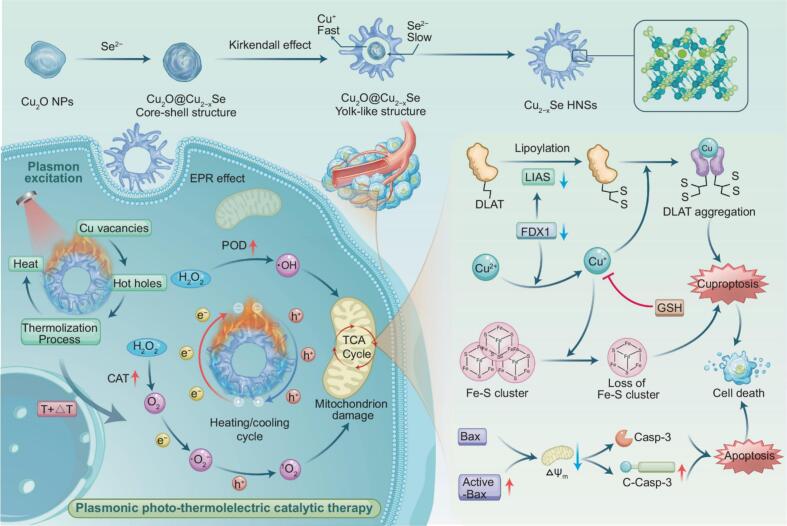
Illustration of the synthesis process of Cu_2-x_Se HNSs, and anti-tumor process inducing cuproptosis and apoptosis activated by synergistic photothermal, enzymatic, and plasmonic thermoelectric catalysis therapies. Adapted with permission from ref. (Yang et al., 2024a), copyright 2024, Springer Nature.

The copper-deficient structure of Cu_2-x_Se also enables novel thermoelectric catalytic therapy. Yang et al. (Yang et al., 2024a) reported a high-performance plasmonic-thermoelectric catalytic nanoplatform based on urchin-like copper-deficient Cu_2-x_Se hollow nanospheres (HNSs). Under 1064 nm laser irradiation, the nanospheres exhibit intense plasmonic absorption and an exceptionally high PCE of 67.0%, generating a pronounced temperature gradient that drives a potent thermoelectric catalytic effect, producing cytotoxic ROS. Density functional theory (DFT) calculations reveal that copper vacancies critically enhance this process by elevating charge carrier concentration and electrical conductivity. Beyond thermoelectric catalytic therapy (TECT), the robust photothermal effect concurrently boosts the intrinsic peroxidase (POD)-like and catalase (CAT)-like activities of the nanospheres, amplifying ROS production. Furthermore, released Cu ions accumulate intracellularly, inducing cuproptosis via toxic aggregation of mitochondrial proteins. This work establishes a pioneering synergistic mechanism combining ROS-mediated damage and cuproptosis through a singular nanostructure, uniquely enabling dual pathways of mitochondrial dysfunction (Fig. 4).

### 2.3 Hollow Cu_x_O_y_ nanoplatforms

Hollow copper oxide nanomaterials (e.g., Cu_2_O and CuO) represent a burgeoning frontier in cancer theranostics. Their high specific surface area, abundant active sites, and tunable pore structures synergize with the intrinsic Fenton-like activity and TME-responsive degradability for CDT and cuproptosis (Li et al., 2024c; Wang et al., 2022; Wang et al., 2020). Current research advances along three directions such as TME-triggered turn-on systems, immunotherapy synergy, and post-surgical adjuvants.

**Fig. 5:**
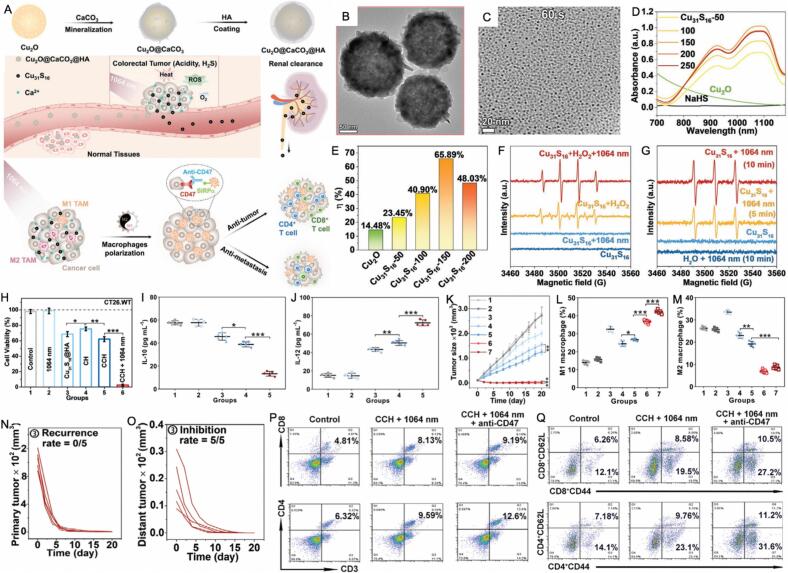
(A) The synthetic route of CCH, the CRC TME-triggered bio-decomposition, anti-tumor responses and renal clearance of CCH, and the antitumor immune responses activated by CRC TME in combination with CD47 blockade. Signal regulatory protein α (SIRPα) is an inhibitory transmembrane receptor highly expressed on macrophages. The interaction between SIRPα and CD47 on tumor cells delivers a “don't eat me” signal, which suppresses macrophage-mediated phagocytosis and facilitates tumor immune escape. The combination of CCH nanoplatform and anti-CD47 antibody can block the CD47-SIRPα signaling pathway. This intervention reverses the immunosuppressive TME, promotes the polarization of tumor-associated macrophages to the M1 phenotype, and increases the infiltration of cytotoxic T lymphocytes, ultimately exerting robust anti-tumor and anti-metastasis effects. (B) TEM image of CCH. (C) The mimetic CRC TME-triggered bio-decomposition of CCH after 60 s. (D) Vis–NIR absorption spectra of Cu_2_O, NaHS, and Cu_2_O with different concentrations (50, 100, 150, 200, and 250 μg mL^−1^) dispersed in 200 μg mL^−1^ of NaHS. (E) The PCE of Cu_2_O with different concentrations (50, 100, 150, 200, and 250 μg mL^−1^) dispersed in 200 μg mL^−1^ of NaHS. (F) The ESR spectra of DMPO/•OH and (G) TEMP/^1^O_2_ after different treatments. (H) The cytotoxicity assessment on CT26.WT cells (pH 6.5). Secretion levels of IL-10 (I) and IL-12 (J) in the supernatant after different treatments. Groups: 1) control, 2) 1064 nm, 3) CH, 4) CCH, 5) CCH + 1064 nm. Data are presented as mean ± SD (*n* = 5). (K) The tumor sizes of BALB/c mice after various treatments. The percentage of M1 (L) and M2 (M) macrophages in the tumors. Groups: 1) control, 2) 1064 nm, 3) anti-CD47, 4) CH, 5) CCH, 6) CCH + 1064 nm, 7) CCH + 1064 nm + anti-CD47. (N, O) The primary and distant tumor sizes after different treatment with CCH + 1064 nm + anti-CD47. (P) The percentage of CD8^+^ and CD4^+^ T cells in distant tumors after different treatments. (Q) The determination of effector memory cell (CD44^+^CD62L^−^) and central memory T cells (CD44^+^CD62L^+^) in CD8^+^ T cells and CD4^+^ T cells in spleens. Data are presented as mean ± SD (n = 5). (*p < 0.05, **p < 0.01, and ***p < 0.001). Adapted with permission from ref. (Chang et al., 2020), copyright 2020, Wiley-VCH GmbH.

To leverage the acidic, hydrogen sulfide (H_2_S)-rich colorectal cancer (CRC) microenvironment, Chang et al. (Chang et al., 2020) developed a core-shell nanoconstruct (CCH) via in situ mineralization of calcium carbonate (CaCO_3_) shell onto hollow mesoporous Cu_2_O, followed by hyaluronic acid (HA) coating for tumor targeting (Fig. 5A, B). In acidic conditions, CaCO_3_ dissolves, exposing Cu_2_O to H_2_S and converting it into NIR-absorbing Cu_31_S_16_ nanocrystals, dramatically enhancing PCE from 14.48% to 65.89% (Fig. 5C-E). The resulting Cu_31_S_16_ nanocrystals also demonstrated potent ROS generation, producing •OH in the presence of H_2_O_2_ and singlet oxygen (^1^O_2_) under 1064 nm laser irradiation, enabling combined CDT and PDT (Fig. 5F, G). This H_2_S-triggered turn-on of synergistic PTT/PDT/CDT together with calcium overload-mediated therapy resulted in significant CT26.WT cancer cell death (Fig. 5H). The treatment also modulated the immunosuppressive TME, evidenced by increased IL-12 and decreased IL-10 (Fig. 5I, J). For in vivo application, an anti-CD47 antibody was integrated to block the “don't eat me” signal on cancer cells, enhancing phagocytosis and T-cell immunity. The combination of CCH, 1064 nm laser, and anti-CD47 led to complete tumor ablation (Fig. 5K). Tumor-associated macrophages (TAMs) analysis confirmed an immune-favorable shift, with M1 macrophages rising to 42.2% and M2 macrophages falling to 8.7% (Fig. 5L, M). This localized treatment ignited a systemic anti-tumor immune response, inducing a potent abscopal effect. Primary tumors were eradicated, distant metastatic growth was suppressed, and no recurrence occurred within 21 days (Fig. 5N, O). Analysis of distant tumors showed infiltration of cytotoxic CD8^+^ and helper CD4^+^ T cells (Fig. 5P). Furthermore, increased levels of central and effector memory T cells in the spleen suggested the establishment of durable immune memory against recurrence and metastasis (Fig. 5Q). In summary, the TME-responsive CCH nanoplatform, combined with CD47 blockade, effectively reprograms the immunosuppressive TME and elicits a potent, systemic anti-tumor immune response for CRC treatment.

Hu et al. (Hu et al., 2025a) constructed an injectable thermosensitive hydrogel (CMCI Gel: CuMnOx@CuO_2_@IR820) to minimize systemic toxicity. The gel incorporates three components: (1) hollow CuMnO_x_ nanozymes derived from cubic Cu_2_O through a redox reaction with the strong oxidant potassium permanganate (KMnO_4_) and an Oswald ripening process, which exhibit pH-regulated multienzyme activity, (2) copper peroxide (CuO_2_) nanoflowers as an exogenous H_2_O_2_ source, and (3) the PTA IR820. Within acidic microenvironments, the gel simultaneously generates ROS and enables low-temperature PTT (< 48 °C). This dual action enhances enzymatic activity and promotes controlled hydrogel dissolution. As a result, CMCI Gel demonstrates broad-spectrum antibacterial efficacy, ablates residual tumor cells, mitigates inflammation, and promotes angiogenesis and wound healing, addressing post-operative recurrence and infection.

### 2.4 Hollow copper silicate nanoplatforms

Hollow copper silicate nanoplatforms are typically synthesized via a hydrothermal method with SiO_2_ spheres as sacrificial templates (Peng et al., 2021; Ren et al., 2024). They combine the catalytic and cuproptosis-inducing advantages of copper-based materials with the high stability, good biocompatibility, and tunable porosity of silicate-based materials. This unique combination has spurred extensive research into synergistic cancer therapy. Additionally, the bioactive properties of copper silicate make it suitable for promoting tissue regeneration following tumor ablation, addressing the critical need for functional recovery in post treatment healing.

Liu et al. (Liu et al., 2019a) reported cancer cell membrane-coated mesoporous Cu/Mn silicate nanospheres (mCMSNs) for novel cancer theranostics. The coating enables homotypic targeting, while the nanospheres relieve hypoxia via H_2_O_2_-catalyzed O_2_ generation, enhancing 635 nm laser-induced PDT. GSH-triggered degradation releases Cu^+^/Mn^2+^, depletes GSH, and promotes •OH production via Fenton-like reactions. This dual ROS generation disrupts antioxidant defenses, effectively inhibiting tumor growth. Released Mn^2+^ also enables real-time tumor MRI, integrating diagnosis with enhanced CDT/PDT in one TME-responsive nanoplatform.

**Fig. 6:**
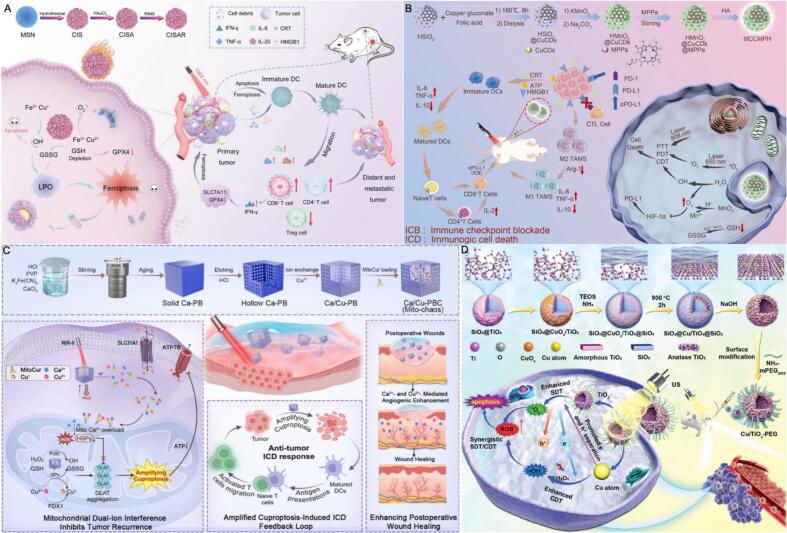
(A) Schematic illustration of CISAR synthesis with immunotherapy and ferroptosis co-enhanced therapeutic mechanism. Adapted with permission from ref. (Du et al., 2022), copyright 2022, Wiley-VCH GmbH. (B) Synthesis of MCCMPH and the mechanism of antitumor. Adapted with permission from ref. (Yu et al., 2025), copyright 2025, Elsevier Inc. (C) Preparation of the mito-chaos nanoplatform and its application in antitumor, wound healing and tissue regeneration. Adapted with permission from ref. (Du et al., 2025), copyright 2025, American Chemical Society. (D) Schematic diagram of the synthetic process of single-atom Cu/TiO_2_-PEG nanosonosensitizers by a reformative wrap-bake-strip method, and synergetic-enhanced ROS generation of single-atom Cu/TiO_2_-PEG nanosonosensitizers for sono/chemo-dynamic therapeutic process under US irradiation against TNBC. Adapted with permission from ref. (Chen et al., 2023), copyright 2023, Wiley-VCH GmbH.

Du et al. (Du et al., 2022) fabricated a closed-loop Au-modified hollow Cu/Fe silicate nanoplatform (CIS@Au/R848, CISAR) (Fig. 6A). Hollow CIS templates were synthesized via hydrothermal Kirkendall evolution driven by sodium borohydride (NaBH_4_)-mediated Si—O cleavage, allowing Au nanoparticle in-situ growth and high R848 immunoadjuvant loading. Plasmonic Au and semiconductor Cu endowed CISAR outstanding photothermal capacity. Tumor acidity and laser-triggered hyperthermia synergistically amplify its CDT-relevant Fenton-like •OH generation while intensifying intracellular GSH consumption. On CT26 cells, laser treatment induced robust ROS burst and GSH exhaustion, suppressing GPX4 to accumulate lipid peroxides (LPO) and activate ferroptosis. The treatment triggered ICD with increased calreticulin exposure, high mobility group box 1 (HMGB1) release and secretion of pro-inflammatory TNF-α and IL-6. In vivo, CISAR combined with 1064 nm laser completely ablated primary tumors and suppressed distant lesions via strong abscopal effects, accompanied by massive intratumoral CD4^+^/CD8^+^ T cell infiltration. CD8^+^ T cell-derived IFN-γ downregulated tumoral SLC7A11 to block GSH synthesis and further amplify ferroptosis, supported by lowered GPX4 and SLC7A11 expression. H&E, TUNEL and Ki-67 staining verified severe tumor damage and suppressed proliferation.

### 2.5 Hollow copper ferrite nanoplatform

Hollow copper ferrite nanostructures exemplify a sophisticated bimetallic platform that unites the therapeutic versatility of copper with the catalytic and magnetic properties of iron. This integration enhances CDT through amplified Fenton/Fenton-like activity driven by abundant Fe^3+^/Fe^2+^ redox couples. Simultaneously, Cu contributes high PCE for PTT and induces cuproptosis. Critically, interfacial interactions between the two metals can establish a self-amplifying catalytic cycle, where Cu^+^/Cu^2+^ redox cycling promotes Fe^2+^ regeneration, sustaining ROS production (Hu et al., 2024a; Liu et al., 2019b). These hollow bimetallic systems often exhibit improved biocompatibility and more predictable TME-responsive degradation compared to single-metal analogues.

Chu et al. (Chu et al., 2023) designed a multifunctional nanoplatform based on polydopamine (PDA)-coated hollow mesoporous copper ferrite nanoparticles (HCFP NPs) for synergistic CDT/PTT/chemotherapy. HCF NPs were synthesized via a solvothermal method using Pluronic F127 as a soft template. Subsequently, an NIR-responsive PDA shell was deposited on the surface, followed by DOX loading to form DOX@HCFP. Within the acidic TME, DOX@HCFP decomposes, releasing Cu^2+^, Fe^3+^, and DOX. The released metal ions deplete GSH while being reduced to Fe^2+^ and Cu^+^, which catalyze H_2_O_2_ into highly toxic •OH, amplifying CDT efficacy. Simultaneously, the PDA shell exhibits a high PCE of 35.25%, elevating local temperature for PTT while accelerating ROS generation and enhancing DOX uptake. In vitro and in vivo studies demonstrate that this trimodal strategy effectively suppresses osteosarcoma progression.

Hollow inorganic Cu-based nanostructures establish a robust foundation for cancer theranostics, offering intrinsic photothermal, catalytic, and cuproptosis-inducing functions. Their high physiological stability and well-defined synthesis make them suitable for applications requiring durable nanostructures under biological conditions. Nevertheless, hollow cavity volume, shell thickness and cavity-to-shell ratio vary drastically across Kirkendall effect, Ostwald ripening and template-assisted fabrication routes, directly governing drug loading efficiency, molecular diffusion rate and TME-responsive degradation kinetics. The Kirkendall effect relies on differential diffusion rates between core and shell materials, creating void space at the nanoscale. DOX/Mn-Cu_2-x_Se@AC registers a specific surface area of 44.267 m^2^/g and a DOX encapsulation efficiency of 28.36%. Co_3-x_Cu_x_S_4_ delivers a specific surface area of 21.94 m^2^/g with an average size of ∼250 nm. The dense walls of these Kirkendall-derived hollow structures impede molecular permeation, yielding slow, near-linear release kinetics and moderate degradation rates. Ostwald ripening involves the dissolution of small inner crystallites and redeposition on larger outer crystals, creating hollow interiors through mass relocation. Cu_2_O@CaCO_3_@HA exemplifies this pathway: the thick, compact carbonate shell severely restricts solute efflux, suppressing burst release almost entirely. Degradation in simulated TME proceeds at a conspicuously slow pace, extending circulation half-life at the cost of delayed Cu^+^/Cu^2+^ liberation and attenuated catalytic activity. Template-assisted methods, including hard or soft templating strategies, enable independent, precise tuning of cavity size and shell thickness. CMSNs present a ∼ 30 nm cavity, ∼30 nm shell, specific surface area of 247.9 m^2^/g, pore volume of 0.684 cm^3^/g, and pore size of 5.16 nm. CISA affords an ∼85 nm hollow architecture with a specific surface area of 342 m^2^/g and an R848 encapsulation efficiency of 21.7%. DOX@HCFP delivers a 275 nm diameter, ∼80 nm shell, specific surface area of 33.013 m^2^/g, pore size of ∼3.5 nm, and maximum DOX loading capacity of ∼63.6%. The thin mesoporous shell facilitates rapid molecular penetration, producing a characteristic biphasic release profile: an initial fast burst followed by sustained cargo outflow. Despite tunable structural properties achieved via different synthetic pathways, the fixed composition and limited surface chemistry of pure inorganic matrices still restrict further functional diversification, such as integrating complex organic molecules or precisely modulating degradation kinetics. To overcome these limitations while retaining hollow architectures, researchers have developed a modular doping strategy.

## 3 Hollow Cu-doped nanoplatforms for cancer treatment

Hollow Cu-doped nanoplatforms are synthesized by incorporating copper ions into the matrix of hollow nanomaterials, including carbon, Prussian blue (PB), and titanium dioxide (TiO_2_). This strategy preserves the inherent advantages of the hollow matrix such as high cargo-loading capacity and TME responsiveness, while introducing copper-specific therapeutic functions. Furthermore, synergistic interactions between copper and the host matrix can enhance therapeutic efficacy and reduce systemic toxicity. This section categorizes these nanoplatforms by matrix material and discusses their design principles, mechanisms, and preclinical applications.

### 3.1 Hollow Cu-doped carbon nanoplatforms

Hollow carbon nanomaterials such as carbon spheres and carbon dots (CDs) exhibit excellent PCE, high chemical stability, and good biocompatibility (Algarra et al., 2025; Singh et al., 2025). Doping Cu into hollow carbon matrices enhances their catalytic activity and facilitates novel therapeutic synergies. Consequently, a major focus lies in designing systems for combined PTT and immunotherapy to elicit robust systemic antitumor responses.

Tao et al. (Tao et al., 2023) constructed a hollow iron/copper-modified carbon-based nanozyme (HCS FeCu) that utilizes mild photothermal heating to potentiate pyroptosis mediated immunotherapy. Possessing multi enzyme mimetic activity, HCS FeCu induces pyroptosis upon light irradiation via a ROS dependent axis involving Tom20, Bax, Caspase 3, and GSDME cleavage. Beyond direct tumoricidal activity, this process stimulates ICD and reprograms the TME. In vivo studies demonstrate that combining HCS FeCu induced pyroptosis with anti PD 1 markedly enhances systemic antitumor immunity and suppresses tumor progression. Theoretical simulations clarify that mild hyperthermia facilitates high energy electron generation and promotes surface activated molecular oxygen, synergistically augmenting ROS yield. Collectively, this work presents an effective strategy to reverse tumor immunosuppression by activating pyroptosis, offering a rational design paradigm for synergistic photothermal-immunotherapeutic nanoplatforms.

Yu et al. (Yu et al., 2025) reported an “in situ synthesis-template etching” strategy to fabricate hyaluronic acid (HA)-functionalized hollow manganese dioxide (HMnO_2_) nanocarriers for co-encapsulating copper-doped carbon dots (CuCDs) and the photosensitizer methyl pyropheophorbide a (MPPa). The synthesis involves forming CuCDs within a hollow mesoporous silica (HSiO_2_) template, followed by MnO_2_ coating and silica etching. This design ensures permanent physical entrapment of CuCDs within the HMnO_2_ cavity, resolving premature detachment issues. Upon HA-mediated CD44 targeting, the HMnO_2_ shell decomposes in the acidic TME, generating O_2_ to alleviate hypoxia and downregulate the HIF-1α/PD-L1 axis. Encapsulated CuCDs serve as dual-functional agents for CDT and PTT, depleting GSH and synergizing with MPPa PDT to amplify ROS and robust ICD. This initiates a systemic antitumor immune response, stimulating DC maturation, reprogramming TAMs from M2 to M1, and promoting CD4^+^/CD8^+^ T cell infiltration. In a dual-tumor model, MCCMPH combined with anti-PD-L1 significantly inhibited both primary and distant tumors. This study exemplifies the potential of structurally integrated hollow Cu-doped carbon nanoplatforms to synergistically combine microenvironment modulation, multimodal therapeutics, and immune activation for achieving potent and systemic anticancer outcomes (Fig. 6B).

### 3.2 Hollow Cu-doped PB nanoplatforms

PB and its analogs are widely used in cancer theranostics due to their excellent photothermal properties, MRI contrast ability, and Fenton-like catalytic activity (Yan et al., 2025; Zhang et al., 2025a). Hollow-structured PB is typically synthesized through acid etching (Tao et al., 2025). Doping Cu into hollow PB nanoplatforms enhances their cuproptosis-inducing ability and enables synergistic cuproptosis/ferroptosis therapy, which is particularly effective for overcoming apoptotic resistance in cancer cells.

Zhang et al. (Zhang et al., 2024) rationally designed a copper-doped hollow Prussian blue (CHP) co-loaded with indocyanine green (ICG) and oxygenated perfluorohexane (O_2_-PFH@CHPI) for cooperative cuproptosis/ferroptosis induction. In the TME, the Cu-doped nanozyme depletes GSH and catalyzes Fenton-like reactions to accumulate ROS. Under NIR irradiation, photothermal heating accelerates catalysis and triggers O_2_ release, enhancing PDT and oxidative stress. This environment activates cuproptosis via Cu^+^-mediated DLAT aggregation and Fe—S cluster loss, while simultaneously promoting lipid peroxidation and GPX4 inactivation to drive ferroptosis. The two pathways synergistically disrupt mitochondrial metabolism, forming a self-reinforcing therapeutic cycle.

Du et al. (Du et al., 2025) engineered a mitochondria-targeted “chaos” inducer (Mito-chaos) by integrating a curcumin derivative (Mito-Cur) into a hollow PB structure co-doped with calcium and copper (Ca/Cu-PBC NPs). This design exploits the hollow architecture for high-capacity ion loading and the inherent NIR-II photothermal property of PB for spatiotemporal activation. The nanoplatform achieves precise mitochondrial co-delivery of Cu^2+^ and Ca^2+^ ions. Within the organelle, Cu^2+^ induces classic cuproptosis through DLAT aggregation, while Ca^2+^ influx provokes mitochondrial overload and disrupts adenosine triphosphate (ATP) production, potently amplifying cuproptotic cell death. Moreover, Cu-doping confers POD- and GPx-like activities. Under 1064 nm irradiation, strong NIR-II photothermal conversion intensifies these catalytic reactions, accelerating ROS generation and DAMP release, culminating in robust ICD and systemic antitumor immunity. Notably, Mito-chaos also facilitates post-surgical tissue repair: released Cu^2+^ stimulates angiogenesis and collagen deposition, while Ca^2+^ promotes hemostasis, simultaneously addressing residual melanoma and enhancing wound healing (Fig. 6C).

### 3.3 Hollow Cu-doped TiO_2_ nanoplatforms

TiO_2_ is a well-known sonosensitizer and photosensitizer widely used in SDT and PDT, but its efficacy is limited by low charge separation efficiency and poor ROS generation (Liu et al., 2025a; Zhu et al., 2025). To overcome these limitations, Chen et al. (Chen et al., 2023) engineered a single-atom copper-doped hollow TiO_2_ nanosonosensitizer (Cu/TiO_2_) for synergistic CDT/SDT against triple-negative breast cancer (TNBC). The innovation lies in atomic-level doping of single Cu atoms into titanium vacancies within the hollow TiO_2_ framework. HAADF-STEM confirms uniform Cu dispersion, while XRD excludes crystalline Cu phases and EDS rules out Cu—N_4_ coordination. DFT calculations reveal that each Cu atom bonds with four adjacent lattice oxygen atoms to form a Cu—O_4_ structure, with XPS confirming the coexistence of Cu^+^ and Cu^2+^. Charge density analysis shows that Cu atoms capture free electrons and inhibit the recombination of US-generated electron–hole pairs, while the Cu doping introduces mid-gap energy levels that lower the excitation energy and enhance sonodynamic efficiency. Meanwhile, the reversible Cu^+^/Cu^2+^ redox cycle of the Cu—O_4_ sites confers outstanding peroxidase-like activity for CDT. Upon US activation, Cu/TiO_2_ initiates mutually reinforcing nanodynamic therapy, wherein SDT and CDT synergistically amplify ROS generation. Both in vitro and in vivo evaluations demonstrated significant inhibition of TNBC growth with high biosafety. This work establishes a transformative paradigm, illustrating how atomic-level copper integration can convert conventional hollow sonosensitizers into high-performance, multifunctional agents for non-invasive cancer treatment (Fig. 6D).

Cu doped nanoplatforms demonstrate how strategic Cu introduction into foreign matrices expands functional versatility, enabling improved charge separation for enhanced catalysis or combined cuproptosis/ferroptosis. However, doping typically relies on atomic or cluster level dispersion, which may constrain the amount of bioavailable Cu and limit loading of larger Cu based nanoagents. For applications requiring high dose copper delivery or integration of pre-formed multifunctional copper NPs, a matrix supported architecture offers a compelling alternative.

## 4 Hollow matrix-supported Cu-based nanoplatforms for cancer treatment

Hollow matrix-supported Cu-based nanoplatforms are constructed by loading or anchoring Cu-based therapeutic agents onto hollow matrix materials such as hollow silica NPs, MOFs, and iron oxide nanoflowers (IONFs). The hollow matrix serves as a carrier to improve stability, biocompatibility, and cargo-loading capacity. Moreover, the matrix can be engineered to respond to TME cues, enabling controlled release of Cu^2+^/Cu^+^ and therapeutic payloads.

### 4.1 Hollow silica-supported Cu-based nanoplatforms

Hollow mesoporous organosilica nanoparticles (HMONs) and hollow mesoporous silica nanoparticles (HMSNs) are widely used as carriers for Cu-based agents due to their large pore volume, tunable surface chemistry, and good biocompatibility (Cheng et al., 2019; Huang et al., 2018; Li et al., 2020). They are typically synthesized via a “structural difference-based selective etching” strategy using SiO_2_ as a sacrificial hard template (Huang et al., 2024). HMON-supported Cu-based nanoplatforms integrate copper's therapeutic functions with the drug delivery capability of HMONs, enabling synergistic combination therapy.

**Fig. 7:**
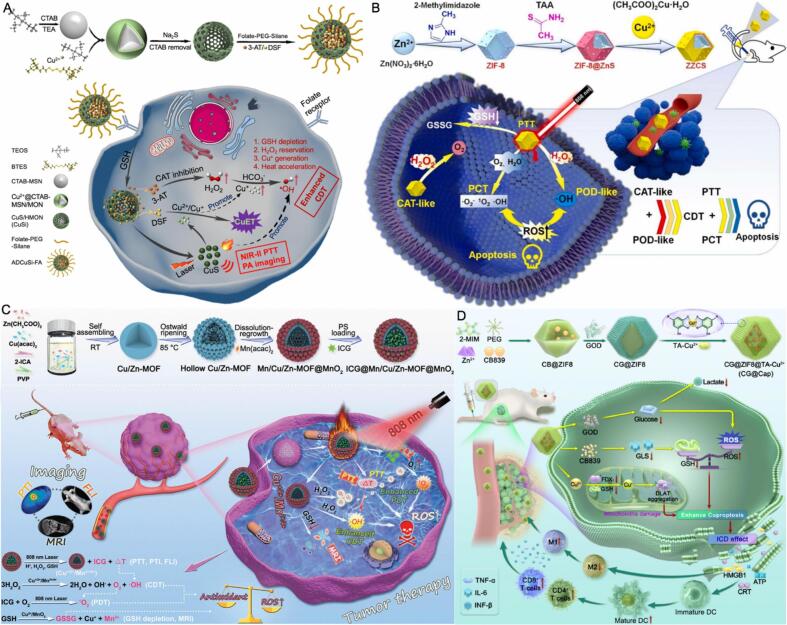
(A) Schematic illustration of the preparation of ADCuSi-FA and its application in PA imaging and synergistic CDT/NIR-II PTT/chemotherapy. Adapted with permission from ref. (Zhang et al., 2025c), copyright 2025, Elsevier Inc. (B) Schematic representation of the synthesis pathway of ZZCS core-shell nanocages and schematic diagram for CDT, PCT and PTT. Adapted with permission from ref. (Chen et al., 2025), copyright 2025, Elsevier Inc. (C) Schematic illustration for the fabrication of ICG@Mn/Cu/Zn-MOF@MnO_2_ and its therapeutic mechanism for imaging guided ROS-augmented synergistic PTT/PDT/CDT. Adapted with permission from ref. (Cheng et al., 2021), copyright 2024, Wiley-VCH GmbH. (D) Schematic illustration of the preparation of CG@Cap and its mechanisms in tumor immunometabolic therapy. Following intravenous injection, CG@Cap accumulates at the tumor site and is internalized by tumor cells. The released CB839 and GOD effectively inhibit glutamine metabolism and glycolysis, leading to energy deprivation and redox imbalance within the cells. This disruption enhances Cu-induced cuproptosis and simultaneously triggers ICD. Subsequently, damage-associated molecular patterns such as CRT, HMGB1, and ATP are released, thereby eliciting a robust antitumor immune response that effectively suppresses tumor growth. Adapted with permission from ref. (Li et al., 2025a), copyright 2025, American Chemical Society.

Our group (Zhang et al., 2025c) developed a novel ultrasmall CuS nanodots-embedded and folate-modified HMON nanoplatform co-loaded with 3-amino-1,2,4-triazole (3-AT) and DSF (ADCuSi-FA). Upon GSH-responsive intracellular release in 4 T1 cancer cells, 3-AT suppressed catalase activity, preserving endogenous H_2_O_2_ to promote •OH production through Cu^+^-mediated Fenton-like reactions. Meanwhile, DSF chelated Cu^2+^ to enhance chemotherapeutic efficacy, and the in situ generated Cu^+^ further amplified CDT. Additionally, the CuS nanodots facilitated NIR-II PTT and PAI, with the induced hyperthermia accelerating CDT efficiency. This synergistic PTT/chemotherapy boosted-CDT nanoplatform, featuring H_2_O_2_ preservation and GSH depletion, exerted significant tumor growth suppression (Fig. 7A). Wu et al. (Wu et al., 2018) grafted ultrasmall Cu_2-x_Se onto HMONs via disulfide linkers, loading DOX into the hollow interior (DOX@HCu). Upon focused US-mediated blood–brain barrier opening, the platform achieved targeted brain delivery. Cu_2-x_Se enables PAI and renal clearance, while the reductive TME triggers biodegradation and on-demand drug release, significantly inhibiting orthotopic tumor growth. Ma et al. (Ma et al., 2022) developed cisplatin-loaded HMONs sealed with a bimetallic Zn/Cu-MOF gatekeeper (Pt@HMOS@ZC). In the acidic TME, the MOF degrades to release Pt and Cu^2+^. Released Cu^2+^ depletes GSH, sensitizing cells to Pt and catalyzing •OH generation for CDT. Zhu et al. (Zhu et al., 2024a) designed a pH-responsive dual-delivery system using tannic acid (TA)/Cu^2+^-coated HMSNs to co-encapsulate DSF and Cu^2+^, enabling selective cancer cell eradication through combined chemotherapy and CDT. HMSNs were also used to load a Cu(II)-doped polydopamine complex (HMSNs@PDA-Cu) for synergistic PTT/CDT (Zhang et al., 2021). PDA acts as a PTA with enhanced conversion efficiency, while its SOD-like activity supplies H_2_O_2_ for Cu-mediated Fenton-like •OH generation. Localized PTT heat further accelerates •OH production, creating a self-amplifying loop for tumor eradication.

### 4.2 Hollow MOF-supported CuS nanoplatforms

MOFs are porous crystalline materials with high surface area and tunable composition, making them promising carriers for Cu-based agents (Jia et al., 2026; Kabir et al., 2025; Liu et al., 2025b). Among various MOFs, zeolitic imidazolate framework-8 (ZIF-8) has attracted widespread attention due to its excellent biocompatibility, pH-responsive degradation, and high drug loading capacity (Gao et al., 2025; Hu et al., 2025b; Zhang et al., 2025b).

Chen et al. (Chen et al., 2025) constructed a hollow core-shell nanocage based on a ZIF-8 template, denoted ZIF-8@ZnS@CuS (ZZCS), for combined therapy. Cu^2+^ ions within the CuS shell confer POD-like and CAT-like activities. POD-like activity catalyzes H_2_O_2_ conversion to toxic •OH for CDT while depleting GSH to elevate ROS levels. CAT-like activity decomposes excess H_2_O_2_ into O_2_, alleviating hypoxia and enhancing photocatalytic therapy (PCT). The hollow core-shell heterojunction facilitates excellent NIR absorption and multiple internal light reflections. Upon 808 nm laser irradiation, ZZCS yields a high PCE (78.1%) for effective PTT and simultaneously generates •OH, superoxide anions (•O_2_^−^), and ^1^O_2_ through photocatalytic processes, enabling synergistic tumor elimination. This integrated nanoplatform enables the synergistic elimination of tumor cells by combining hyperthermia with a multi-faceted oxidative assault (Fig. 7B).

### 4.3 Hollow IONF-supported CuS nanoplatforms

IONFs exhibit excellent magnetic hyperthermia (MHT) performance and MRI contrast (Benassai et al., 2024; Nicolás-Boluda et al., 2020), loading CuS onto hollow IONFs enables integration of MHT, PTT, and PDT. Curcio et al. (Curcio et al., 2019) developed a tri-therapeutic nanohybrid (IONF@CuS) featuring a γ-Fe_2_O_3_ nanoflower core for MHT and a spiky CuS shell. Synthesized via a facile aqueous template-sacrificial route, the hybrid exhibits a PCE of 42 ± 6%, a specific absorption rate of ∼350 W g^−1^ under magnetic stimulation, and significant ROS generation. In vivo, PTT alone achieved complete tumor regression at low copper doses. The nanoplatform defines operational windows for each modality individually and in combination, and supports dual-modal PAI/MRI. By integrating dual heating (MHT + PTT) with PDT, IONF@CuS enables synergistic tumor attack with reduced dosage and treatment intensity.

Matrix supported systems excel at shielding and delivering pre-assembled copper nanoagents, separating the roles of carrier and therapeutic. This compartmentalization allows independent optimization of the hollow matrix and copper payload. However, physical encapsulation or surface attachment can sometimes lead to inefficient release or reduced accessibility of active sites. To achieve maximal atomic efficiency and direct integration of copper into the carrier scaffold, the most elegant solution lies in hollow Cu based coordination complexes.

## 5 Hollow Cu-based complexes for cancer treatment

Hollow Cu-based complexes have emerged as a promising category in cancer theranostics, complementing inorganic, doped, and matrix-supported systems. They offer greater flexibility in structural design and functional integration, allowing for more precise regulation of therapeutic mechanisms, enhanced specificity, and reduced systemic toxicity. This section focuses on three main types: hollow Cu-based MOFs, hollow Cu-TA-based nanoplatforms, and other emerging hollow Cu-based complexes.

### 5.1 Hollow Cu-based MOFs

Hollow Cu-based MOFs, constructed by coordinating copper ions with organic linkers, combine high porosity and large surface area with the hollow structure's cargo-loading advantage (Li et al., 2023a; Wang et al., 2025; Yu et al., 2024). These nanoplatforms efficiently encapsulate various therapeutic agents and integrate multiple modalities, making them ideal for synergistic theranostics. The adjustable coordination environment of copper enables precise regulation of its redox properties, optimizing Fenton-like catalytic activities.

Cheng et al. (Cheng et al., 2021) developed a hollow mixed-valence (Cu^+^/^2+^) Cu/Zn-MOF via Ostwald ripening for trimodal imaging and synergistic therapy. Heating in the presence of manganese(II) acetylacetonate (Mn(acac)_2_) introduced Mn^2+^/MnO_2_, yielding a mixed-metal, mixed-valence (Cu^+^/^2+^/Mn^2+^/^4+^) structure. The hollow framework encapsulates ICG, enabling PTT and fluorescence imaging under laser irradiation while generating ^1^O_2_ for PDT. Concurrently, Cu^+^/Mn^2+^ catalyze Fenton-like reactions with endogenous H_2_O_2_ to produce •OH for CDT. Cu^2+^ and MnO_2_ deplete GSH, amplifying oxidative stress, while Mn^2+^ provides turn-on MRI contrast. H_2_O_2_ decomposition alleviates hypoxia, enhancing PDT, and localized PTT heat accelerates Fenton reactions, creating potent drug-free synergy (Fig. 7C).

Using an innovative unsaturated coordination-etching integration (UCEI) strategy, Xu et al. (Xu et al., 2022) fabricated a copper/iron hybrid hollow amorphous metal-organic framework (HaMOF) co-loaded with DOX. The nanoplatform amplifies oxidative stress by catalytically converting H_2_O_2_ to •OH while depleting GSH. It concurrently disrupts copper/iron homeostasis by downregulating metal exporters (ATP7A and ferroportin-1), trapping both metals in the cytosol. The resultant Cu overload induces authentic cuproptosis, while excess Fe^2+^/Cu^2+^ fuels Fenton-driven lipid peroxidation and ferroptosis. Combined with DOX-induced apoptosis, this triple-death-pathway synergy achieves near-complete tumor eradication.

### 5.2 Hollow Cu-TA-based nanoplatforms

Tannic acid (TA), a natural polyphenol, exhibits excellent metal-coordinating capacity through its abundant hydroxyl groups, enabling construction of Cu-TA-based nanoplatforms. These platforms benefit from TME-responsive properties and good biocompatibility, making them versatile carriers for targeted delivery and in situ drug activation while minimizing off-target toxicity (Guo et al., 2021; Zhang et al., 2025f). TA's intrinsic acidity can also etch pH-sensitive nanomaterials such as ZIF-8, providing a straightforward route to generate hollow nanostructures.

Meng et al. (Meng et al., 2021) constructed a pH-responsive nanocomposite (DSF/DOX@ZIF-8@Cu-TA) with a ZIF-8 core (loaded with DSF and DOX) and a Cu-TA shell. In the acidic TME, released Cu^2+^ chelates with DSF to form cytotoxic bis(diethyldithiocarbamate)‑copper complex (CuET) in situ, while generated Cu^+^ catalyzes Fenton-like ROS production. CuET synergistically enhances DOX efficacy via ROS-MAPK/NF-κB pathways, yielding superior outcomes through a “nontoxicity-to-toxicity” transition.

Li et al. (Li et al., 2025a) developed a hollow dual-starvation nanocapsule (CG@Cap) integrating immunometabolic therapy with copper-induced cell death. The nanoplatform is fabricated via one-pot coordination-driven assembly: glucose oxidase (GOD) is adsorbed onto ZIF-8 NPs preloaded with glutamine inhibitor CB839, followed by in situ formation of a TA- Cu^2+^ complex shell. The acidity of TA simultaneously etches the ZIF-8 core, generating a hollow structure and encapsulating cargo in a single step. Following internalization, CG@Cap initiates a two-stage cascade. First, co-released GOD and CB839 block glycolysis and glutamine metabolism, disrupting energy supply and redox equilibrium. This metabolic perturbation primes cells for cuproptosis induced by Cu^2+^ liberated from the shell. Subsequent ICD and metabolic reprogramming engage a self-reinforcing loop: ICD stimulates antitumor immunity amplified by TME immunostimulation, while metabolic inhibition mitigates tumor resistance to cuproptosis. By harnessing TA's dual roles as a pH-sensitive etchant and metal-chelating ligand, this design directly couples targeted metabolic interference with in situ cuproptosis induction, eliciting a potent systemic antitumor response (Fig. 7D).

### 5.3 Hollow CuET-based nanoplatforms

In addition to hollow Cu-based MOFs and Cu-TA-based nanoplatforms, other emerging hollow Cu-based complexes have also attracted increasing attention. A novel hollow nanoplatform, ART@CuT/ETH HNP, was developed using a chelation competition-induced hollowing (CCIH) strategy (Xu et al., 2023b). This approach simultaneously creates a CuET complex while etching a copper-based template to form a hollow cavity. The structure is loaded with artemisinin (ART) and functionalized with a hyaluronan-based shell for enhanced circulation and tumor targeting. Upon accumulation in the TME, the nanoplatform responds to acidic and GSH-rich conditions, triggering a triple-amplified oxidative stress cascade. Released Cu^2+^ catalyzes ART to generate carbon-centered radicals and facilitates Fenton-like conversion of H_2_O_2_ to •OH. The disulfide-rich shell depletes GSH, further disrupting redox homeostasis. This amplified oxidative stress sensitizes cancer cells to cuproptosis (via DLAT oligomerization and mitochondrial dysfunction), while concurrently promoting ferroptosis (via GPX4 inhibition) and triggering apoptosis (via CuET-mediated ubiquitinated protein accumulation). This synergistic modality integrates cuproptosis, ferroptosis, and apoptosis, exemplifying how advanced nanocarrier design can orchestrate multiple cell-death pathways for potent anticancer therapy.

Notably, the mechanistic framework underlying this concurrent induction of cuproptosis and ferroptosis is shared across multiple hollow Cu-based nanoplatforms. The crosstalk begins with GSH depletion as the primary convergence hub: GSH removal deprives GPX4 of its essential cofactor, permitting lipid peroxide accumulation and ferroptosis, while loss of copper chelation liberates Cu^+^ to drive DLAT aggregation and Fe—S cluster loss (LIAS↓), triggering cuproptosis. This process is amplified by a ROS burst, which further exacerbates both DLAT aggregation and LPO. At the metabolic level, the copper–iron redox cycle sustains Fenton chemistry, while Fe—S cluster loss impairs mitochondrial respiration (NADH↓, α-KG↑). Both pathways ultimately converge on mitochondria, resulting in ΔΨm collapse and ATP depletion. The ensuing mitochondrial dysfunction releases additional ROS, which further deplete GSH and intensify both death signals, thereby establishing a self-amplifying feedback loop. This cooperative mechanism underpins the potent antitumor efficacy of hollow Cu-based nanoplatforms that concurrently activate these two death pathways.

Copper ions coordinated with organic ligands form the porous, hollow framework—blurring the line between carrier and cargo. Materials such as hollow Cu-MOFs and Cu-TA assemblies inherit the excellent cargo-loading capacity of hollow structures while ensuring that every Cu atom is potentially bioactive and accessible. This fully integrated paradigm unifies hollow architecture, stimulus-responsive degradation, and therapeutic copper centers into a single chemical entity, offering unparalleled design precision for synergistic theranostics.

## 6 Conclusions and perspectives

Hollow Cu-based nanoplatforms represent a paradigm shift in cancer theranostics. Their unique architectural blueprint provides an unparalleled combination of high therapeutic payload capacity, large functionalizable surface area, and TME-responsive behavior. More than passive carriers, these nanostructures are dynamic systems that actively participate in therapeutic processes. Their degradable nature ensures precise, on-demand release of co-delivered agents and bioactive Cu^2+^/Cu^+^ for enhancing Fenton-like reactions and cuproptosis. Critically, these mechanisms, alongside PTT, PDT, and chemotherapy, robustly trigger ICD. By converting the tumor into an in situ vaccine, hollow Cu-based systems reprogram the immunosuppressive TME, enabling synergistic combinations with immunotherapies to achieve durable systemic antitumor immunity and suppress metastasis. This review has surveyed advances across four major categories: inorganic Cu nanostructures, Cu-doped composites, matrix-supported Cu systems, and Cu-based coordination complexes. Key properties of representative nanoplatforms are systematically summarized in Table 1. Despite remarkable advances, several challenges must be addressed to bridge the gap between laboratory innovation and clinical application.

**Table 1:** Summary of various hollow Cu-based nanoplatforms for cancer treatment.

Material	Mechanism	Synthetic steps	Tumor model	Biomedical application	Reference
Cu_2-x_S@ES@LA	Kirkendall effect	4	CT26	PTT/CDT/Cuproptosis/Immunotherapy	(Cheng et al., 2025)
Co_3-x_Cu_x_S_4_	Kirkendall effect	4	4 T1	PTT/CDT	(Jiang et al., 2022)
TPZ@Cu-SnS_2-x_/PLL	Kirkendall effect	4	4 T1	PTT/PDT/CDT/Chemotherapy	(Zhu et al., 2024b)
DOX/Mn-Cu_2-x_Se@AC	Kirkendall effect	5	H-22	PTT/CDT/Chemotherapy	(Yin et al., 2025)
Cu_2-x_Se HNSs	Kirkendall effect	3	CT26	PTT/CDT/TECT/Cuproptosis	(Yang et al., 2024a)
Cu_2_O@CaCO_3_@HA	Ostwald ripening process	3	CT26	PTT/PDT/CDT/Chemotherapy/Calcium overload/Immunotherapy	(Chang et al., 2020)
CuMnO_x_@CuO_2_@IR820	Redox reaction and Oswald ripening process	4	4 T1	PTT/PDT/CDT	(Hu et al., 2025a)
mCMSNs	Self-sacrificing template method	4	MCF-7	PDT/CDT	(Liu et al., 2019a)
CISA	Self-sacrificing template method	4	CT26	PTT/CDT/Ferroptosis/Immunotherapy	(Du et al., 2022)
DOX@HCFP	Soft template method	4	143b	PTT/CDT/Chemotherapy	(Chu et al., 2023)
HCS-FeCu	Hard template and pyrolysis-etching method	5	4 T1	PTT/Enzymatic therapy/Immunotherapy	(Tao et al., 2023)
MCCMPH	In situ synthesis-template etching method	4	CT26	PTT/PDT/CDT/Immunotherapy	(Yu et al., 2025)
O_2_-PFH@CHPI	Acid etching method	5	Huh7	PTT/PDT/CDT/Ferroptosis/Cuproptosis	(Zhang et al., 2024)
Mito-chaos	Acid etching method	4	B16	PTT/CDT/Cuproptosis/Calcium overload /Immunotherapy	(Du et al., 2025)
Cu/TiO_2_	Wrap-bake-strip method	6	4 T1	CDT/SDT	(Chen et al., 2023)
ADCuSi-FA	Structural difference-based selective etching method	5	4 T1	CDT/PTT/Chemotherapy	(Zhang et al., 2025c)
DOX@HCu	Structural difference-based selective etching method	6	U87	Chemotherapy	(Wu et al., 2018)
Pt@HMOS@ZC	Structural difference-based selective etching method	5	A549	CDT/Chemotherapy	(Ma et al., 2022)
DSF-AHMSN@TA-Cu	Structural difference-based selective etching method	4	HCT-116	CDT/Chemotherapy	(Zhu et al., 2024a)
HMSNs@PDA-Cu	Structural difference-based selective etching method	2	MCF-7	PTT/CDT	(Zhang et al., 2021)
ZZCS	Kirkendall effect	3	4 T1	PTT/CDT/PCT	(Chen et al., 2025)
IONF@CuS	Kirkendall effect	3	PC3	PTT/Magnetic hyperthermia/PDT	(Curcio et al., 2019)
ICG@Mn/Cu/Zn-MOF@MnO_2_	Ostwald ripening process	4	U87	PTT/PDT/CDT	(Cheng et al., 2021)
ZCProP	Chelation competition-induced hollowing	2	4 T1	Cuproptosis/Ferroptosis	(Deng et al., 2024)
DOX@Fe/CuTH	Unsaturated coordination-etching integration	4	4 T1	CDT/Chemotherapy/Cuproptosis/Ferroptosis	(Xu et al., 2022)
DSF/DOX@ZIF-8@Cu-TA	TA-induced ZIF-8 decomposition	3	MDA-MB-231	CDT/Chemotherapy	(Meng et al., 2021)
CG@Cap	TA-induced ZIF-8 decomposition	3	SCC7	CDT/Starvation therapy/Cuproptosis/Immunotherapy	(Li et al., 2025a)
ART@CuT/ETH	Chelation competition-induced hollowing	3	4 T1	CDT/Chemotherapy/Cuproptosis/Ferroptosis	(Xu et al., 2023b)

This review comprehensively surveys the burgeoning landscape of hollow Cu-based nanoplatforms including inorganic, doped, matrix-supported, and complex-based hollow structures for cancer treatment.

Safety assessment must move beyond acute toxicity to evaluate chronic exposure, organ-specific accumulation, and metabolic fate of released ions and nanomaterial residues. The impact of degradation by-products on copper homeostasis, neural function, and systemic inflammation requires careful investigation. Cardiomyocytes, hepatocytes, and neurons maintain constitutively high mitochondrial respiratory flux and abundant lipoylated proteins, rendering them intrinsically vulnerable to sustained intracellular copper overload if unregulated Cu ion release occurs. However, the TME-responsive hollow Cu nanostructures feature acid/GSH-labile thin shells that remain structurally intact under the neutral, low-GSH physiological environment of healthy organs, avoiding premature Cu leakage; only within acidic, GSH-enriched tumor lesions do hollow carriers disassemble and locally liberate Cu species to initiate cuproptosis. Tumor cells exhibit elevated intracellular copper levels due to overexpression of copper importers (CTR1) and impaired efflux transporters (ATP7A/B), creating a state of copper dependency that normal cells do not share. In vivo biodistribution and histopathological assays of hollow Cu ferrite and Cu chalcogenide nanoplatforms further validate negligible organ toxicity: no obvious tissue damage, inflammatory infiltration or pathological lesions were detected in heart, liver and kidney after therapeutic dosing, confirming the tissue-selective therapeutic window of hollow Cu nanosystems. Validation in larger animal models (e.g., primates) will be essential to predict human pharmacokinetics and toxicology. Targeting precision can be improved by incorporating active targeting ligands (peptides, antibodies, aptamers) that recognize overexpressed tumor markers. Organelle-specific targeting (mitochondria, lysosomes, nucleus) can dramatically enhance efficacy by delivering Cu^2+^/Cu^+^ or ROS-generating agents directly to critical compartments, lowering required doses and reducing off-target effects. Synergy optimization requires deeper exploration of true synergistic mechanisms where one modality actively enhances another (Mao et al., 2025; Luo et al., 2026). For instance, the photothermal effect of CuS can ablate tumors, improve intratumoral blood flow, potentiate CDT catalytic reactions, and promote drug penetration. Future work should focus on feedback-enabled systems where CDT-generated ROS or cuproptosis-induced stress further sensitizes tumors to subsequent treatments. Optimizing the sequence, timing, and dosage of combined stimuli will be key. To facilitate clinical translation, scalable and reproducible manufacturing is a prerequisite. Multi-step templating routes, such as those relying on the Kirkendall effect or galvanic replacement, already present inherent challenges in batch-to-batch reproducibility and precise control over cavity volume, shell thickness, and porosity. Two specific processing steps warrant particular attention for industrial scale-up. High-temperature calcination or pyrolysis demands inert atmospheres, prolonged processing times, and stringent temperature uniformity, which collectively escalate energy costs and introduce variability that is difficult to control at larger scales. Acid etching, meanwhile, is not only time-consuming and generates corrosive waste, but also critically influences shell thickness and cavity dimensions, parameters that directly dictate drug loading and release kinetics. The high sensitivity of these structural features to etching time, temperature, and acid concentration renders uniform control exceptionally challenging in commercial production, potentially leading to significant batch-to-batch variation. Developing simpler, greener, and more robust synthetic methods, such as one-pot synthesis or continuous flow manufacturing, will therefore be essential for translating these promising nanoplatforms into clinically and commercially viable products. Synthesis-property relationships must be systematically mapped to fine-tune size, degradation kinetics, and cargo-release profiles. Clinical translation is supported by the modularity of hollow Cu-based nanoplatforms for personalized medicine. Integrating real-time imaging with therapeutic response monitoring could enable adaptive, patient-specific regimens. Focused investigational new drug-enabling studies should be initiated—selecting a lead candidate for a well-defined cancer type, establishing robust quality-control metrics, and conducting comprehensive toxicology and efficacy studies in clinically relevant models.

In summary, hollow Cu-based nanoplatforms have reshaped cancer nanomedicine by unifying diagnostics, multimodal therapy, and immune modulation within a single biodegradable entity. Through interdisciplinary collaboration spanning materials chemistry, pharmacology, immunology, and clinical oncology, they hold promise to transition from preclinical concepts to transformative clinical tools for precision oncology.

## CRediT authorship contribution statement

**Shuang Liang:** Writing – original draft, Investigation, Conceptualization. **Xingran Li:** Writing – review & editing. **Li Zhang:** Writing – review & editing, Writing – original draft, Funding acquisition, Conceptualization. **Wenzhen Zhu:** Writing – review & editing, Supervision, Funding acquisition, Conceptualization. **Shaoyan Gan:** Writing – review & editing, Writing – original draft, Conceptualization.

## Declaration of competing interest

The authors declare that they have no known competing financial interests or personal relationships that could have appeared to influence the work reported in this paper.

## Data Availability

No data was used for the research described in the article.
